# Laxity index measurement on stress radiographs obtained using the Vezzoni-modified Badertscher distension device technique: repeatability and reproducibility in a large cohort of dogs

**DOI:** 10.3389/fvets.2025.1675958

**Published:** 2025-11-07

**Authors:** Sara Sassaroli, Francesco Gallorini, Francesco Roggiolani, Alberto Salvaggio, Rosario Vallefuoco, Andrea Pratesi, Mario Fordellone, Elisa Campagnoli, Angela Palumbo Piccionello

**Affiliations:** 1 School of Biosciences and Veterinary Medicine, University of Camerino, Camerino, Italy; 2 Technevet, Matelica, Italy; 3 Clinica Veterinaria San Silvestro, Castiglion Fiorentino, Italy; 4 Policlinico Veterinario Gregorio VII, Rome, Italy; 5 Department of Small Animal Surgery, Pride Veterinary Referrals, Derby, United Kingdom; 6 Private Practitioner, Padova, Italy; 7 Unit of Medical Statistics, Department of Mental and Physical Health and Preventive Medicine, University of Campania, Napoli, Italy

**Keywords:** laxity index, repeatability, reproducibility, canine hip dysplasia, stress radiograph

## Abstract

**Introduction:**

The Vezzoni-modified Badertscher distension device (VMBDD) technique is a radiographic method used to assess hip joint laxity, and it is widely used across Europe. While the intra-observer and inter-observer variability of the laxity index (LI) measured on stress radiographs obtained using the VMBDD technique has been reported, it has never been evaluated in a large cohort of patients. The study aims to assess the repeatability and reproducibility of the LI measured on stress radiographs obtained using the VMBDD technique in a large cohort of dogs.

**Methods:**

Stress radiographs obtained using the VMBDD method were analyzed for medium to large breed dogs, aged between 4.5 and 6 months and presented between 2021 and 2024 for screening of hip dysplasia. The LI for each hip was blindly measured by three observers with different levels of experience. Significant intra- and inter-observer variability was evaluated to assess the repeatability and reproducibility of the LI, respectively. Statistical testing was performed, and a *p*-value of <0.05 was considered statistically significant. Inter-observer and intra-observer intraclass correlation coefficients (ICCs) were evaluated.

**Results:**

A total of 195 stress radiographs (390 hip joints) were included. The inter-observer ICC showed moderate agreement (ICC = 0.55, 95% CI 0.50–0.59). Estimated marginal means (EMMeans) indicated that Observer 3 consistently provided higher LI values compared to Observers 1 and 2 across all time points (e.g., at T1: 0.484 vs. 0.410 and 0.438, *p* < 0.001 for Observer 1 vs. Observer 3). The repeatability within each observer was excellent for all three observers (Observer 1: ICC = 0.94, 95% CI 0.93–0.96; Observer 2: ICC = 0.99, 95% CI 0.99–0.99; Observer 3: ICC = 0.95, 95% CI 0.94–0.96).

**Conclusion:**

In-house evaluation of the LI on stress radiographs obtained using the VMBDD technique showed that it was a highly repeatable procedure but a moderate reproducible measurement due to a systematic upward bias by an observer with less experience. Nevertheless, the mean differences could be considered negligible in a clinical setting due to their low impact on the definitive diagnosis.

## 1 Introduction

Canine hip dysplasia (CHD) is a common developmental disease affecting medium, large, and giant breed dogs. It is a complex multifactorial disease influenced by both genetic (hereditary and polygenic) and environmental factors, leading to secondary osteoarthritis and subsequent clinical signs of discomfort, disability, lameness, and pain (1–4).

Henricson et al. (5) describe CHD as a disease that originates from a “varying degree of laxity of the hip joint permitting subluxation during early life, giving rise to varying degrees of shallow acetabulum and flattening of the femoral head, finally inevitably leading to osteoarthritis”. Hip joint laxity is reported to be a significant predisposing factor for the development of hip osteoarthritis, and it is the first clinical and radiographic finding in dogs predisposed to develop CHD once skeletally mature (1, 6).

Radiography is a diagnostic imaging tool commonly used for the detection of CHD. Radiographic screening for CHD is performed worldwide based on the evaluation of standard ventrodorsal hip extended (VD) views, according to three international breeding organizations: the Fédération Cynologique Internationale (FCI), the Orthopedic Foundation for Animals (OFA), and the British Veterinary Association and the Kennel Club (BVA/KC) (7, 8). This screening method is not suitable for assessing the risk of developing hip dysplasia in young dogs. Jassen and Spurrel reported that at 6, 12, and 24 months of age, 16–32%, 63–69%, and 92–95% of dogs examined were accurately diagnosed as dysplastic based on the VD radiographic assessment conducted up to 5 years of age, respectively (9). For this reason, the OFA has set the earliest age for canine hip screening at 24 months, while the FCI and BVA/KC have established a minimum age of 12 months, with a minimum error in diagnosis of 30% (2, 7).

Early diagnosis of CHD is based on a clinical orthopedic evaluation and radiographic examination using both static and dynamic views, aimed at detecting prodromic findings of the disease (1, 6). Unlike the VD radiographic view, where hip extension results in articular capsule torsion that partially hides hip laxity (10, 11), the stress radiographic method has shown high sensitivity in detecting joint laxity (10–13). Dorsolateral subluxation scores (12), the subluxation index (13), and the Pennsylvania Hip Improvement Program (PennHIP) method (10) quantify hip laxity radiographically.

Over the last 30 years, the PennHIP has been a well-investigated and standardized method (14, 15). Hip laxity is expressed using the distraction index (DI), which quantifies the femur head lateral displacement from the acetabulum (10). The PennHIP method is popular in the United States, but it is not widespread in other parts of the world, probably due to the expensive mandatory training and official PennHIP report, evaluation costs, and the obligation toward digital radiography (16, 17).

The Vezzoni-modified Badertscher distension device (VMBDD) was proposed in Europe as an alternative in-house technique. The VMBDD method was described for the first time by Badertscher in 1990 and modified in 1998 by Vezzoni (18–21). Recent studies have investigated the reliability of this method to assess the hip joint and the interchangeability of the results with the PennHIP method (17–19, 22). The laxity index (LI), analogous to the DI, expresses joint laxity and yields results similar to the PennHIP-based DI and comparable interobserver agreements (17, 18, 22–25).

The scientific literature reports satisfactory technical repeatability and reproducibility of the VMBDD technique and recommends it as a reliable in-house evaluation method for evaluating hip joints in young patients, with a quick and easy learning curve for inexperienced examiners (18, 22). Authors have reported a high inter-observer and intra-observer agreement for LI measurement in a small cohort of dogs (22, 25). The performance of these assessments in a larger group of dogs of different breeds and with a limited age range has not been tested yet.

The study aims to evaluate the intra- and inter-observer variability of the LI measurement in a large cohort of dogs to evaluate its repeatability and reproducibility, respectively. A larger population would allow us to perform a more appropriate statistical analysis and eventually detect different results from those reported so far in the literature.

## 2 Materials and methods

### 2.1 Ethics statement

This study was conducted in compliance with applicable and ethical guidelines and was approved by the Institutional Animal Care and Use Committee of the University of Camerino (protocol no. 4/2025).

### 2.2 Animals

Medium to large breed dogs, aged between 4.5 and 6 months and referred to the Veterinary Teaching Hospital of the University of Camerino (Matelica, Macerata, Italy) and the Veterinary Clinic San Silvestro (Castiglion Fiorentino, Arezzo, Italy) between November 2021 and November 2024 for screening of hip dysplasia were prospectively enrolled in this study. Age, sex, breed, body weight (BW), and body condition score (BCS, 1–9) were recorded. All patients underwent a complete orthopedic examination.

### 2.3 Stress radiograph acquisition

After an anesthetic examination and blood tests confirming unremarkable results, the dogs were premedicated with 3 μg/kg of dexmedetomidine and 0.2 mg/kg of methadone intramuscularly (IM) and induced with 1–4 mg/kg of propofol intravenously (IV) to effect until tracheal intubation. Anesthesia was maintained with 1.2% isoflurane in 100% oxygen during all radiographic evaluations. The radiographic examination includes four radiographic views of the pelvis: VD, frog leg, dorsal acetabular rim (DAR), and stress radiograph (19, 26). For our study, only the stress radiographic view was blindly evaluated.

One stress radiograph per dog was performed by orthopedic surgeons with 3 to 20 years of experience using the VMBDD technique. Despite the experience with the device, the surgeons attended a theoretical-practical course organized by the Veterinary Teaching Hospital of the University of Camerino and the Veterinary Clinic San Silvestro to standardize the stress radiograph technique according to the literature (18, 19, 21).

**Figure 1:**
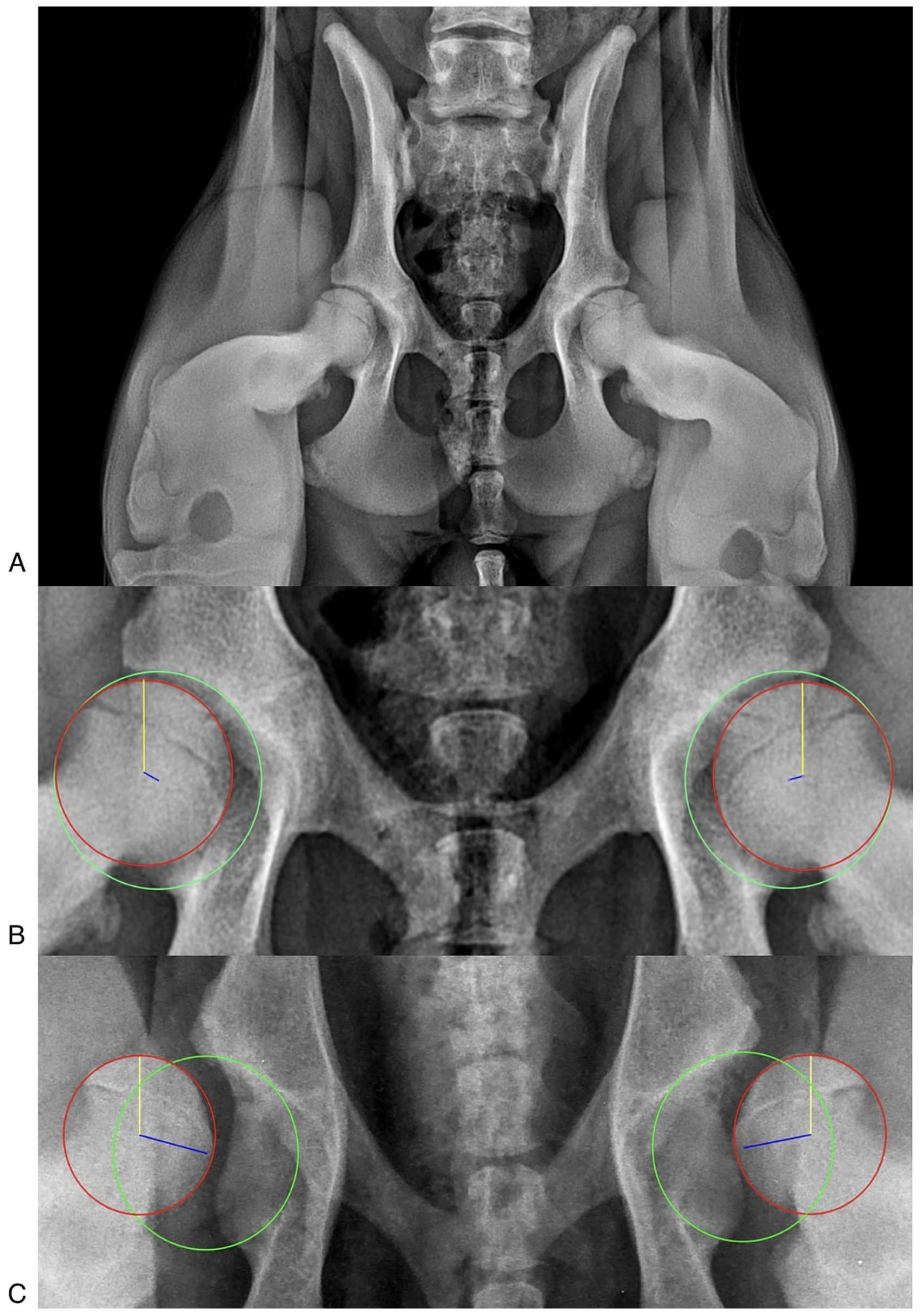
**(A)** Correct stress radiographic view. The obturator foramina and iliac wings are symmetrical, ensuring a straight pelvis. The femurs are symmetrical, and the femoral head is laterally displaced. **(B)** Laxity index (LI) measurement in a patient with an IL < 0.3. The acetabulum was delineated with a circumference passing through the craniolateral acetabular edge, the craniomedial acetabular edge, and the caudolateral acetabular edge (green circumference). Secondly, the head of the femur was delineated with a circumference passing through the cranial and caudal aspects of the head (red circumference). Finally, the distance (*d*, blue line) between the centers of the acetabulum circumference and the femoral head circumference was measured, and the LI was calculated (LI = *d*/*r*), where “*r*” was the radius of the circumference delimiting the femoral head (yellow line). **(C)** LI measurement in a patient with an IL > 0.7.

Stress radiographs with the VMBDD distractor were obtained as previously described (18, 19, 21). Correct positioning and adequate radiographic imaging ensured a straight and symmetrical pelvis (symmetrical obturator foramina and iliac wings), no superimposition of the stifle joint over the hip, symmetric femurs, and lateral displacement of the femoral head, compared to the VD view (Figure 1A).

### 2.4 LI measurement

The DICOM radiographic images were assessed using an open-source medical image viewer (Horos, DICOM viewer, version 3.3.6, Horos Project), and the LI was measured as previously described. The LI was obtained by outlining the acetabulum and the head of the femur with a circumference and measuring the distance (*d*) between the geometric centers of these two circumferences (the geometric center of the acetabulum and the geometric center of the femoral head). The LI was calculated by dividing the distance (*d*) by the radius of the circumference bounding the femoral head (*r*): LI = *d*/*r* (Figures 1B,C) (10, 17, 19, 22, 26).

The LI measurements were blindly performed by three observers with different levels of experience in veterinary orthopedics and hip dysplasia, and all data were recorded using commercial software (Microsoft Excel, Version 16.92, ©2024 Microsoft). The observers included a senior orthopedic surgeon (APP) with more than 20 years of practice and a PhD in orthopedics (Observer 1), a PhD student in orthopedics (SS) with 4 years of practice (Observer 2), and a student of veterinary medicine (EC) (Observer 3). Observer 3 had never performed laxity measurements previously. Therefore, she was mentored by Observers 1 and 2, who taught her in detail all the steps necessary to measure the laxity index.

Each observer independently performed test measurements on 10 hip joints for training purposes. The test measurements of the 10 hip joints were not recorded and included in the study. Subsequently, they blindly measured the LI three times (three measurement sessions) on each hip, with a washout period of 2 weeks between the measurement sessions. All measurements were obtained by each observer in approximately 6 weeks. The left and the right hips were evaluated separately.

### 2.5 Statistical analysis

Continuous variables were reported as either means and standard deviation (SD) or median and interquartile ranges (IQRs) according to their distribution, as assessed by the Shapiro–Wilk normality test. Categorical variables were reported as absolute frequencies and percentages. To explore potential systematic differences among the three observers, an ANOVA test was performed for multiple comparisons within the time points and between the observer groups, followed by a pairwise *t*-test with *p*-values adjusted using the Holm approach. Meanwhile, to assess intra-observer variability, a repeated measures ANOVA test was performed for multiple comparisons between the observer groups and within the time points, followed by a pairwise *t*-test with *p*-values adjusted using the Holm approach. Linear mixed models (LMMs) were used to estimate the longitudinal effects of covariates on the continuous LI scale. The linear mixed effects regression model uses all available data and can properly account for the correlation between repeated measures. The covariates included in the model were the observer (i.e., three different observers), the time point as a categorical variable, and their interaction. For group effect testing, Tukey’s *post hoc* test was performed. Given the limited number of observers (*n* = 3), we modeled the observer as a fixed effect within the LMM framework, together with a random intercept for subjects and the observer to account for repeated measures. This approach allowed us to:

- Adjust for observer-related bias explicitly.
- Test for the effects of observer, time, and their interaction (observer × time) in the same unified framework.

For robustness improvement purposes, inter-observer and intra-observer intraclass correlation coefficients (ICCs) were calculated. All statistical values equal to or less than 0.05 were considered statistically significant. The analysis was conducted using R statistical software (version 4.1.3; 10-03-2022). The calculated LI values were grouped according to dog breeds for the evaluation of breed influence, and the LI for breed groups with more than 20 dogs was subjected to statistical analysis.

## 3 Results

A total of 195 client-owned dogs were enrolled in this study. There were 108 male dogs and 87 female dogs (55.4% male and 44.6% female dog). Their mean ± SD age was 5.2 ± 0.6 months, and their mean ± SD BW was 18.6 ± 5.8 kg, with a mean ± SD BCS of 4.4 ± 0.7 (median, min–max; 4, 3–7).

Assuming *α* = 0.05 (two-sided), power = 0.80, and a small-to-moderate effect size, a total of 195 subjects was required. Using a repeated measures ANOVA approximation with three time points and three observers, the effect size corresponding to this sample size was Cohen’s *f* ≈ 0.20, which translated to a partial *η*^2^ ≈ 0.038 (≈3.8% of variance explained). Then, this effect size was selected to yield the observed sample size of 195 subjects.

A total of 39 breeds were represented, including the following: 33 Golden Retrievers, 30 Border Collies, 29 Labrador Retrievers, 21 German Shepherds, 17 mixed breed dogs, 8 Bernese Mountain dogs, 8 Australian Shepherds, 5 Cocker Spaniels, 5 Cane Corsos, 4 Maremma Sheepdogs, 3 Dobermanns, 3 Rottweilers, 2 English Setters, 2 American Staffordshire Terriers, 1 American Bully, 1 American Pit Bull Terrier, 1 Siberian Husky, 1 Hungarian hound, 1 Bassethound, 1 Bobtail, 1 Czechoslovak Wolf, 1 Kelpie, 1 Apuan Shepherd dog, 1 Shiba Inu, 1 Springer Spaniel, 1 Rhodesian Ridgeback, 1 Pyrenean Mountain dog, 1 Great Dane, 1 Newfoundland dog, 1 Romanian Shepherd, 1 Neapolitan Mastiff, 1 Belgian Shepherd Malinois, 1 Alaskan Malamute, 1 British Staffordshire Terrier, 1 Nova Scotia Retriever, 1 Weimaraner, 1 Lagotto Romagnolo dog, 1 Bavarian Hound, and 1 Caucasian Shepherd. A total of 390 hip joints were analyzed (195 left hip joints and 195 right hip joints).

The mean ± SD of the LI measurements obtained by the three observers is shown in Table 1.

**Table 1:** Mean and standard deviation of the LI measurement.

LI	T1	T2	T3
Observer 1	0.41 ± 0.15	0.41 ± 0.16	0.41 ± 0.15
Observer 2	0.44 ± 0.23	0.43 ± 0.24	0.44 ± 0.23
Observer 3	0.48 ± 0.17	0.45 ± 0.17	0.45 ± 0.17

LI, laxity index; T1, 1° measurement session; T2, 2° measurement session; T3, 3° measurement.

### 3.1 Reproducibility assessment (inter-observer)

**Figure 2:**
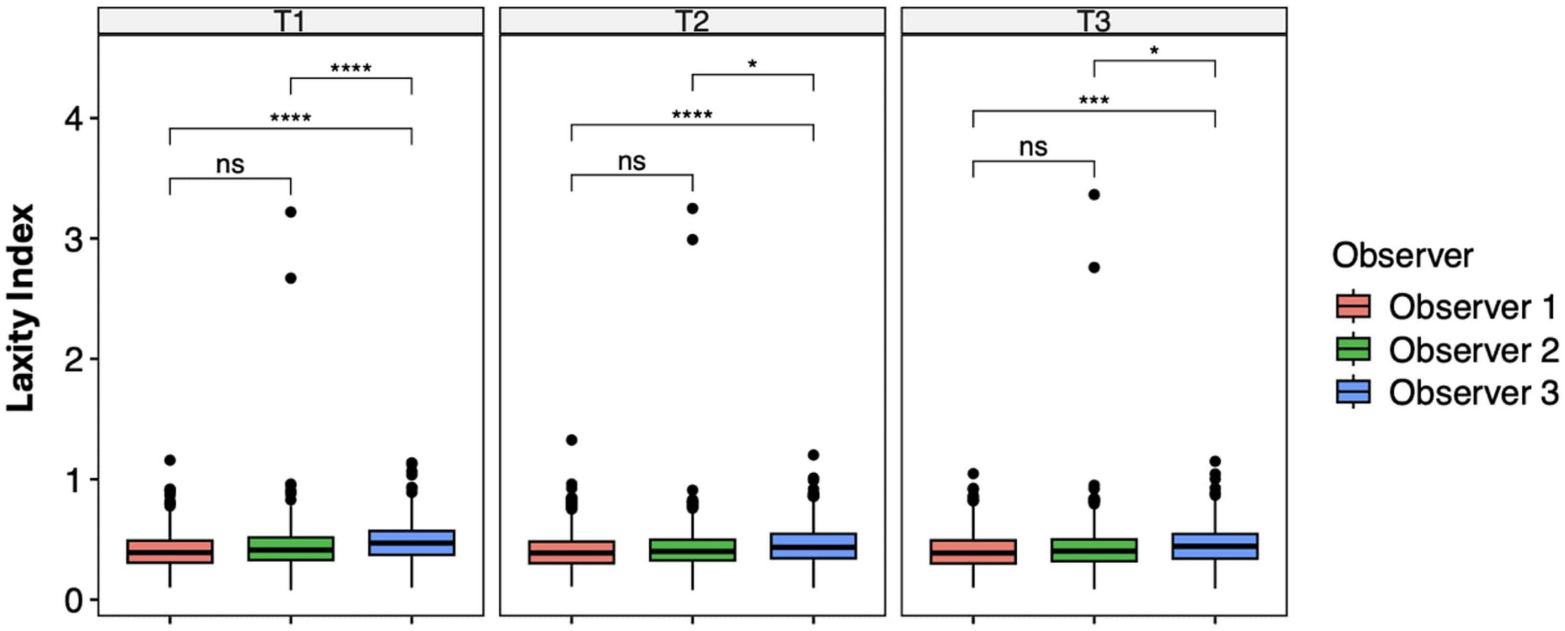
Laxity index distributions across the observer groups at intra-time points. Mean differences were tested using a pairwise *t*-test with Holm’s approach for *p*-value adjustment. An asterisk (*) indicates a significant difference (*p* < 0.05), specifically: ****(*p* < 0.0001), ***(*p* = 0.0001), **(*p* = 0.001), *(*p* = 0.01), and ns (*p* = 0.05 or *p* > 0.05).

The ANOVA test and the pairwise *t*-test with Holm’s approach for *p*-value adjustment revealed significant differences between Observer 1 and Observer 3 (T1, *p* < 0.0001; T2, *p* < 0.0001; T3, *p* = 0.0001) and Observer 2 and Observer 3 (T1, p < 0.0001; T2, *p* = 0.01; T3, *p* = 0.01) at each measurement session. No significant difference was recorded between examiners 1 and 2 (Figure 2).

Reproducibility across the observers was evaluated using both the inter-observer ICC and estimated marginal means (EMMeans) from the mixed-effects model. The inter-observer ICC showed moderate agreement (ICC = 0.55, 95% CI 0.50–0.59). The mixed model with random intercepts for both patients and observers significantly improved the fit compared to a patient-only specification model (ΔAIC = 2472.0; LRT *p* < 0.0001). EMMeans indicated that Observer 3 consistently provided higher LI values compared to Observers 1 and 2 across all time points (e.g., at T1: 0.484 vs. 0.410 and 0.438, *p* < 0.001 for Observer 1 vs. Observer 3). Differences between Observers 1 and 2 were smaller and less consistent, reaching borderline significance at T1–T2, and were not significant at T3. These results confirm that reproducibility across the observers was only moderate, largely due to a systematic upward bias by Observer 3. The highest mean difference between the observers was 0.074 (Table 2).

**Table 2:** The mean difference of the Laxity index between the observer groups at the intra-time point was obtained using a linear mixed model.

Time	Contrast observer	Estimate	*p*-value
T1	Observer 1–Observer 2	−0.0281	0.0595
	Observer 1–Observer 3	−0.0739	<0.0001
	Observer 2–Observer 3	−0.0458	0.0007
T2	Observer 1–Observer 2	−0.0266	0.0805
	Observer 1–Observer 3	−0.0450	0.0009
	Observer 2–Observer 3	−0.0185	0.2939
T3	Observer 1–Observer 2	−0.0253	0.1018
	Observer 1–Observer 3	−0.0456	0.0007
	Observer 2–Observer 3	−0.0203	0.2277

T1, 1° measurement session; T2, 2° measurement session; T3, 3° measurement session.

*p*-value adjustment was performed using the Tukey method to compare a family of three estimates.

### 3.2 Repeatability assessment (intra-observer)

**Figure 3:**
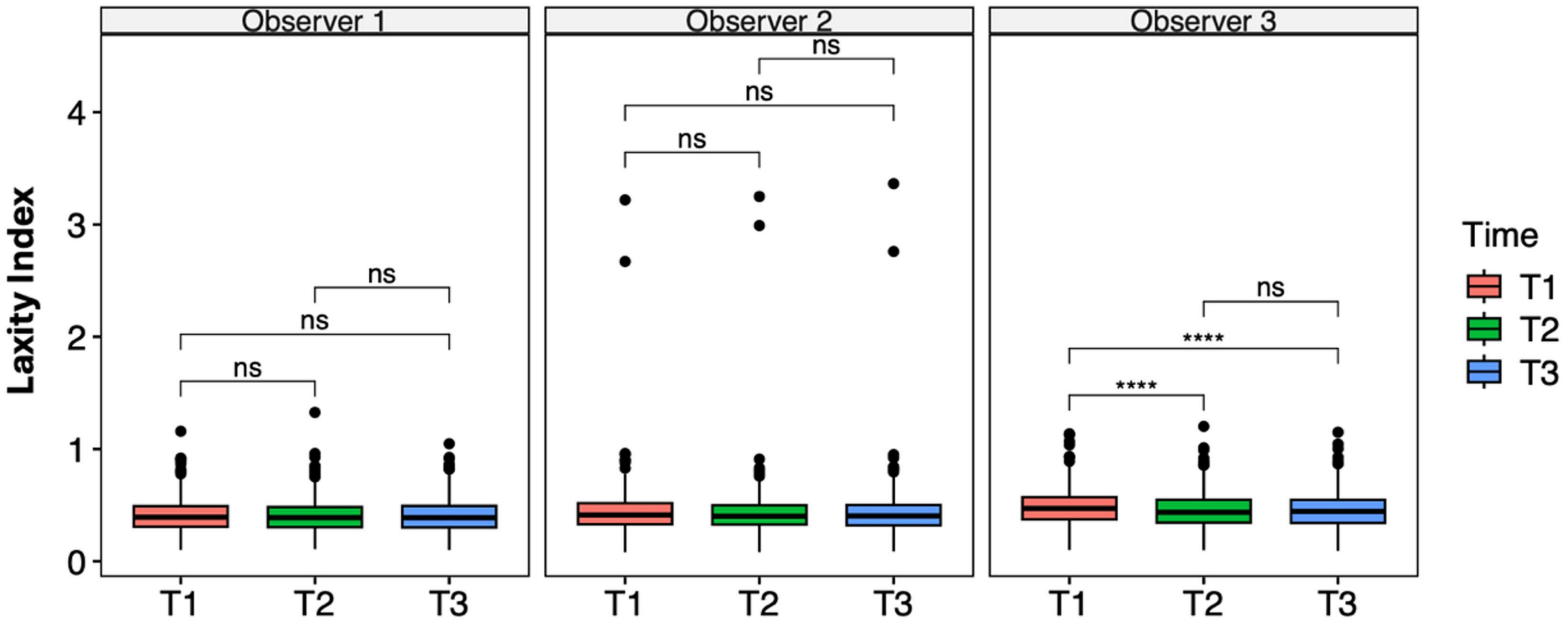
Laxity index distributions across time points within the observer groups. Mean differences were tested using a pairwise *t*-test for paired samples with Holm’s approach for *p*-value adjustment. An asterisk (*) indicates a significant difference (*p* < 0.05), specifically: ****(*p* < 0.0001), ***(*p* = 0.0001), **(*p* = 0.001), *(*p* = 0.01), and ns (*p* = 0.05 or *p* > 0.05).

A significant difference was observed between the first and the second measurement sessions (*p* < 0.001) and the first and the third sessions (*p* = 0.001) in Observer 3, as indicated by the ANOVA test and pairwise *t*-test (Figure 3), consistent with the results from the LMM (Table 3). The mean difference was 0.032 between T1 and T2 and 0.029 between T1 and T3.

**Table 3:** The mean difference of the Laxity index between time points within observers (inter-observer) was obtained using a linear mixed model.

Observer	Contrast (time)	Estimate	*p*-value
Observer 1	T1–T2	0.003	0.947
	T1–T3	0.001	0.990
	T2–T3	−0.002	0.981
Observer 2	T1–T2	0.005	0.861
	T1–T3	0.004	0.904
	T2–T3	−0.001	0.990
Observer 3	T1–T2	0.032	0.001
	T1–T3	0.029	0.002
	T2–T3	−0.003	0.947

T1, 1° measurement session; T2, 2° measurement session; T3, 3° measurement session.

*p*-value adjustment was performed using the Tukey method to compare a family of three estimates.

Repeatability within each observer was assessed using intra-observer ICCs, which were excellent for all three observers (Observer 1: ICC = 0.94, 95% CI 0.93–0.96; Observer 2: ICC = 0.99, 95% CI 0.99–0.99; Observer 3: ICC = 0.95, 95% CI 0.94–0.96). Moreover, EMMeans within each observer were stable across time points (e.g., Observer 1: 0.410–0.409; Observer 2: 0.438–0.434; Observer 3: 0.484–0.455), confirming the absence of meaningful intra-observer variability. These findings demonstrate that each observer was highly consistent in repeated assessments, while reproducibility across the observers remained moderate. The selected model specification, with random intercepts for both the patient and observer, appropriately captured this pattern of high repeatability but only moderate reproducibility.

### 3.3 Reproducibility and repeatability assessment in breeds

Dividing the study population by breed, 4 breed groups with more than 20 dogs were identified: Golden Retriever (GR) group, Border Collie (BC) group, Labrador Retriever (LR) group, and German Shepherd (GS) group. The mean ± SD values for age, BW, BCS, and LI, as well as the sex distribution of groups, are shown in Table 4.

**Table 4:** Means and standard deviation (SD) of age, BW, BCS, gender and LI of difference of GR, BC, LR, and GS groups.

Patient data	GR group	BC group	LR group	GS group
Age (months)	5.1 ± 0.6	5.1 ± 0.4	5.3 ± 0.7	5.0 ± 0.5
BW (kg)	20.1 ± 3.9	13.1 ± 3.2	19.5 ± 3.2	20.6 ± 3.0
BCS	4.6 ± 0.6	4.1 ± 0.4	4.8 ± 0.7	4.1 ± 0.5
Gender	17 M, 16 F	17 M, 13 F	15 M, 14 F	10 M, 11 F
LI	0.47 ± 0.13	0.38 ± 0.31	0.42 ± 0.16	0.40 ± 0.11

BW, body weight; BCS, Body Condition Score; LI, laxity index; GR, Golden Retriever; BC, Border Collie; LR, Labrador Retriever; GS, German Shepherd.

**Figure 4:**
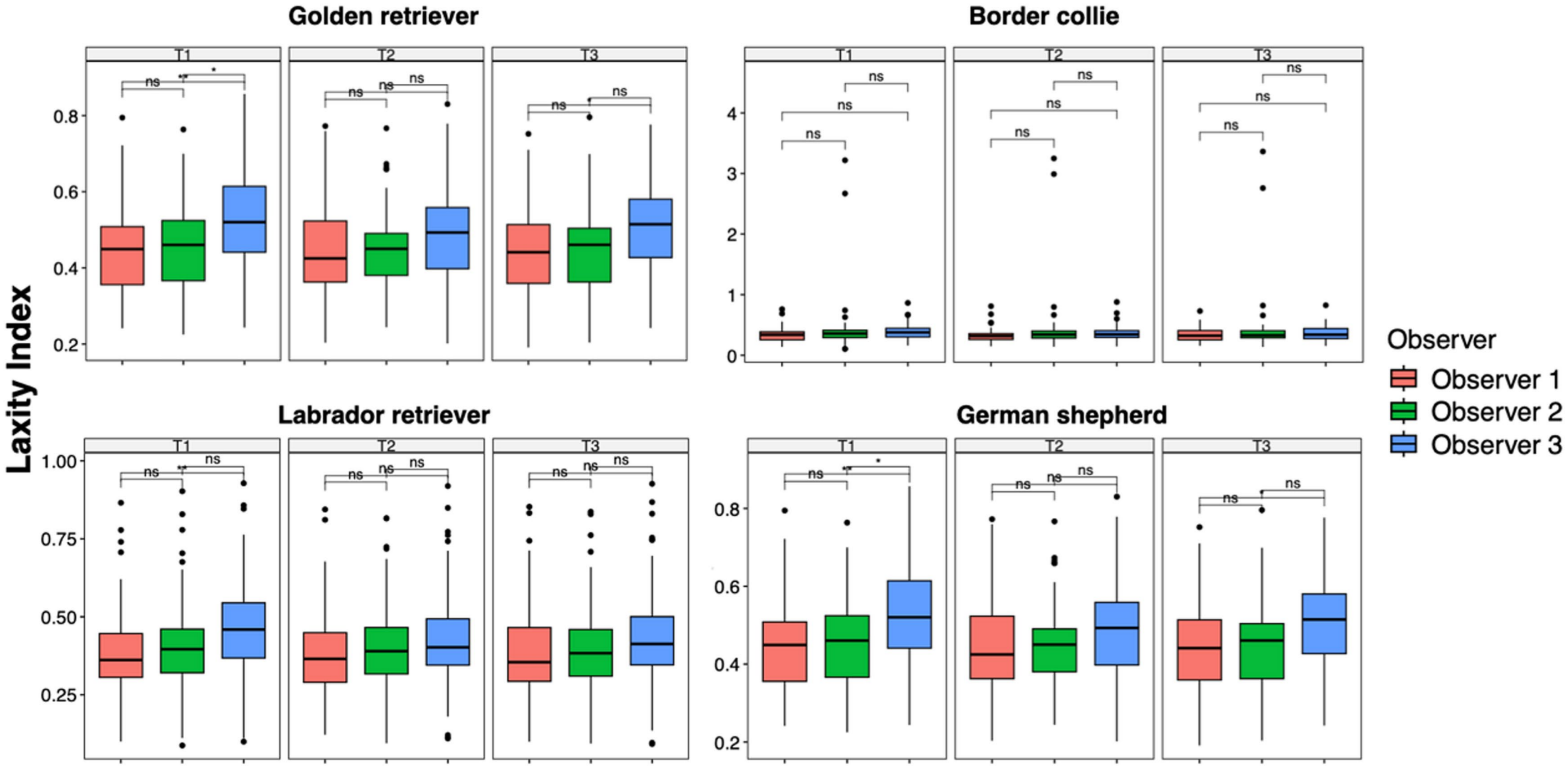
Laxity index distributions across time points within the intra-observer groups for Golden Retrievers, Border Collies, Labrador Retrievers, and German Shepherds. Mean differences were tested using a pairwise *t*-test for paired samples with Holm’s approach for *p*-value adjustment. An asterisk (*) indicates a significant difference (*p* < 0.05), specifically: ****(*p* < 0.0001), ***(*p* = 0.0001), **(*p* = 0.001), *(*p* = 0.01), and ns (*p* = 0.05 or *p* > 0.05).

**Figure 5:**
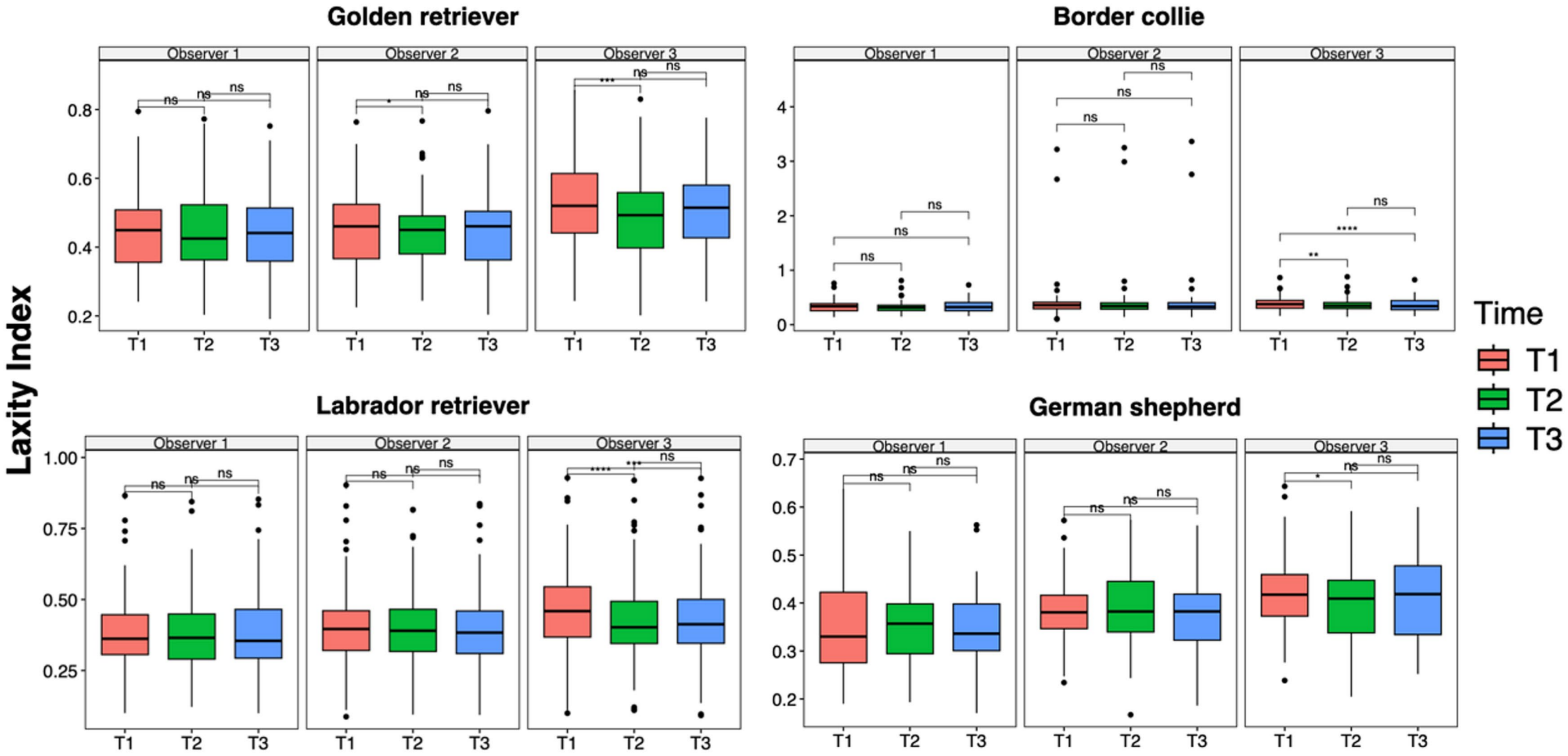
Laxity index distributions across the observer groups at intra-time points for Golden Retrievers, Border Collies, Labrador Retrievers, and German Shepherds. Mean differences were tested using a pairwise *t*-test for paired samples with Holm’s approach for *p*-value adjustment. An asterisk (*) indicates a significant difference (*p* < 0.05), specifically: ****(*p* < 0.0001), ***(*p* = 0.0001), **(*p* = 0.001), *(*p* = 0.01), and ns (*p* = 0.05 or *p* > 0.05).

The results of the ANOVA test and the pairwise *t*-test performed to analyze data between time points for each observer in each breed group were consistent with the analysis previously performed on the entire population. The LI of Observer 3 showed a statistically significant difference between the first and second measurement sessions across all breed groups (GR group, *p* = 0.0001; BC group, *p* = 0.001; LR group, *p* < 0.0001; GS group, *p* = 0.01) and between the first and third sessions in the BC (*p* < 0.0001) and LR groups (*p* = 0.0001). In addition, in the GR group, Observer 2 showed a statistical difference between T1 and T2 (*p* = 0.01) (Figure 4). However, during the first measurement session, significant differences were recorded between Observers 1 and 3 in the GR, LR, and GS groups (*p* = 0.001) and slight but significant differences were recorded between Observers 2 and 3 in the GR and GS groups (*p* = 0.01). During the last measurement session, slight but significant differences were recorded between Observers 1 and 3 only in the GR and GS groups (*p* = 0.01) (Figure 5).

## 4 Discussion

The aim of our study was to assess the intra- and inter-observer variability of the LI measurements in a large cohort of dogs to evaluate the repeatability and reproducibility of the LI. Our data, obtained from a large cohort of various dog breeds compared to published studies (17, 22, 25), showed significant inter-observer variability.

Vidoni et al. (25) reported excellent to good inter-observer agreement for quantitative measurements such as Norberg angle (NA), dorsal acetabular rim angle (DAR), center-edge angle (CEA), and LI; the high level of experience of their observers ensured the accuracy of the measurements. Our study consisted of a more heterogeneous group of observers, with different levels of experience, which led to clinically negligible but statistically significant differences. The student of veterinary medicine (Observer 3) performed the LI measurement for the first time during this study, after personal study and didactic support. Therefore, she was the least experienced operator. Our results differed from previous studies that showed moderate inter-observer agreement (17, 22, 25). However, despite the statistically significant difference, the highest mean difference between the observers was only 0.0739. This value could be considered negligible due to the low impact on the definitive diagnosis because the difference in clinical practice would not be relevant. The literature reports that hip joints with an LI less than 0.4 are not predisposed to develop CHD, while hip joints with an LI higher than 0.7 tend to develop moderate to severe CHD and secondary osteoarthritis. (15, 19). When the LI ranges between 0.4 and 0.7, the development of degenerative joint disease is more influenced by the muscular conformation of the dog and the environment (12, 26, 27). Considering this range of values, the observed higher mean difference between the observers is unlikely to affect the interpretation of the data.

The experience of the examiners could affect the result, and inter-observer agreement could improve with experience (28). Supporting this statement, our results show intra-observer differences between the LI measured by the less experienced operator during the first measurement session and at the other time points. During the next two rounds, Observer 3 improved intra-observer agreement and reduced inter-observer variability. This led to a decrease in significant differences between operators 2 and 3, thereby no difference was detected between these operators at the second time point (T1 *p* < 0.0001 versus T2 *p* = 0.069 and T3 *p* = 0.040). Nevertheless, even in this case, the maximum mean difference was small enough that it would not affect the diagnosis in clinical practice (0.032 ± 0.008). In fact, our results reveal excellent intra-observer agreement, despite the statistical difference observed between measurement times for Observer 3 in the preliminary statistical tests.

The learning curve leading to improvements in intra- and inter-observer variability by the third measurement round appears to have been rapid, given that measurements were conducted at two-week intervals over a total period of 6 weeks. However, the process was also intensive, with 390 measurements performed every 2 weeks contributing to this improvement. Accumulated experience could influence the reproducibility and repeatability of a measurement for several reasons: (1) greater familiarity with the method, reducing errors due to uncertainties or subjective interpretations; (2) development of skill and precision; (3) reduction of subjective variability related to the interpretation of reference points; (4) ability to recognize errors and anomalies; and (5) optimization of workflow, as with practice, the operator becomes more efficient, reducing stress, fatigue, or distractions that may affect measurement accuracy.

The high number of patients enrolled in the study allowed us to further investigate the variability of these measurements considering the different breeds present. Statistical analysis performed in the breed groups reflected the statistical analysis conducted on the entire cohort. It is interesting to note that during each measurement session, the observers did not show statistically significant differences in the group of Border Collies, suggesting high reproducibility. The absence of inter-observer variability in the Border Collie group could indicate the presence of more defined anatomical landmarks in that breed. Another aspect we observed was that this group represented the subgroup of patients with a lower LI compared to other breeds. This may suggest a potential correlation, which warrants further investigation, between the LI value and the reproducibility or repeatability of this measurement. The remaining subgroups partly reflected the results of the general statistical analysis.

**Figure 6:**
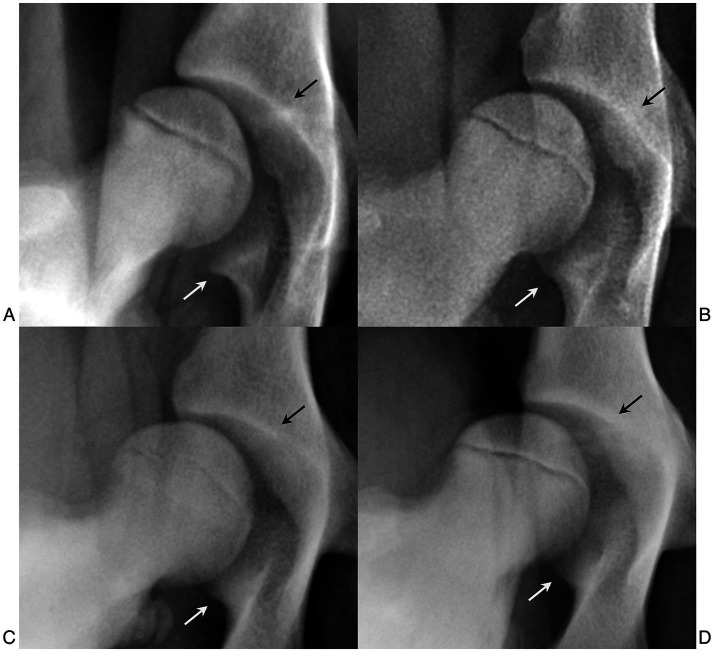
**(A)** Distracted hip joint of a 4.5-month-old female German shepherd. **(B)** Distracted hip joint of a 5-month-old female German shepherd. **(C)** Distracted hip joint of a 6-month-old male Labrador Retriever. **(D)** Distracted hip joint of a 5-month-old male Labrador Retriever. Images **(B–D)** show a caudal pillar (white arrows) that is slightly to moderately less pronounced than in images **(A–C)**. Images **C,D** show a craniomedial acetabular edge (black arrows) that is more defined than in images **(A,B)**.

Our examiners had difficulties in some dogs when outlining the acetabular cavity, particularly in detecting the medial end of the cranial acetabular margin and the caudal pillar of the acetabulum, increasing the risk of intra- and inter-observer variability. These anatomical landmarks affect the position of the center of the acetabular cavity, thereby affecting the distance between it and the center of the femoral head. Difficulties in delineating the acetabulum and the femoral head have been previously reported (22, 29). Further studies are needed to identify possible anatomical differences of the coxofemoral joint in different breeds, in both adult and skeletally immature dogs. These anatomical studies could be interesting not only for the purpose of carrying out these measurements but also for planning the best therapeutic procedures, such as radiographic planning of the acetabular cup for total hip replacement (30–32). From the data we collected, we could not confirm that there were structural differences in the anatomical landmarks needed for the calculation of the LI relative to breed, but there were individual differences that could make these points more or less evident (Figure 6).

In addition, we did not investigate the influence of age on the detection of anatomical landmarks because we enrolled patients with ages ranging from 18 to 24 weeks. The anatomical elements evaluated during the radiographic examination for the early diagnosis of CHD could be more difficult and uncertain to assess before 17 weeks of age (19), and a diagnosis after 24 weeks of age may not be useful for some surgical treatments (juvenile pelvic symphysiodesis) aimed at reducing disease progression (26, 33–37). However, considering that the articular surfaces and joint laxity can change with growth, it would be interesting to evaluate how the patient’s age could influence the judgment of the examining veterinarian for the early diagnosis of CHD. Taroni et al. (38) revealed a significant increase in the DI between 4 and 6 months and a significant decrease between 6 and 12 months, with DI values significantly lower at 12 months than at 4 months. In particular, dogs with a DI > 0.7 at 4 months showed a significant decrease at 6 and 12 months. Furthermore, results published in older studies described a similar but not significant trend, in which the DI increased after 4 months until 6 to 8 months and then decreased at 12 months (16, 27). All these data underline how hip laxity can be influenced by the time of radiographic evaluation.

Until now, the LI has been considered a repeatable and reproducible parameter, useful for obtaining an early diagnosis in young dogs (14, 15, 19, 24). Our study confirms the high repeatability of LI but only moderate reproducibility, due to an upward bias by the observer with less experience. Nevertheless, the highest mean differences revealed were small. This confirms that the LI remains a useful parameter for early diagnosis, particularly when combined with other measurements (19, 24). In fact, although a small risk, there is the possibility that a higher mean reading could result in an overestimation of the LI. For example, if a patient’s LI is measured as 0.35 by one observer and 0.42 by another, this patient could be subjected to unnecessary prophylactic surgical treatment. By interpreting the LI together with other parameters detected during the clinical examination and the early radiographic assessment, this risk is eliminated.

In the literature, there are doubts regarding the LI breed interpretation. In a recent study by Bertal et al. (23), the LI was compared to FCI grading, revealing a moderate-to-good correlation between them: worse FCI grades corresponded to higher LI values, but FCI A and FCI B grades (normal or almost normal hip joint) did not exclude high LI values (min–max, 0.15–0.64 and 0.18–0.75, respectively). Dogs that were phenotypically normal based on FCI grading had a wide range of joint laxity, and the hip joints of some breeds showed more laxity than others. The authors found a slight but significant increase in laxity in the Golden Retrievers compared to the Labrador Retrievers (23). Our data also showed higher laxity in Golden Retrievers and lower laxity in Border Collies of the same age, but these data were not correlated with the diagnosis of CHD or the FCI score, so a statistical comparison of the results and any difference detected would not have provided further information. This observation, however, highlights the need for further investigation into breed-specific LI cutoff values to support an individual approach for each patient.

One limitation of the study is that radiographs were acquired using different radiographic machines, and the literature has reported that, in addition to positioning, image quality may also affect the assessment (10, 22). Therefore, to address this limitation, we used a selection process for the acquired images, and we included in the study only dogs with good image quality and adequate positioning, in accordance with the guidelines (19). Moreover, the LI measurements were performed by a single operator within each observer group with different backgrounds, which may represent a limitation in the interpretation of the results. However, the sample size investigated in this study was substantially larger, and it allowed us to perform a more appropriate statistical analysis. Another limitation is the absence of a comparison between the LI values and the FCI score evaluated in the adult subjects, which did not allow us to provide interesting observations that could help clarify many doubts about the interpretation of the LI.

In conclusion, the LI measurement on radiographic images obtained using the VMBDD technique proved to be highly repeatable, particularly when performed by examiners with moderate or high experience. Furthermore, the LI showed moderate reproducibility in our study due to the differences observed between the less experienced observer and those with high and moderate experience. Nevertheless, the mean differences between the measurements of the observers in each session could be considered negligible in a clinical setting.

Therefore, the LI remains a useful measurement, especially when combined with other parameters, for the early diagnosis of CHD in young dogs. However, it is important to recognize that this parameter may vary depending on the operator performing the measurement and that this variability decreases with the examiner’s level of experience.

## Data Availability

The raw data supporting the conclusions of this article will be made available by the authors, without undue reservation.

## References

[1] Smith GK Mayhew PD Kapatkin AS McKelvie PJ Shofer FS Gregor TP. Evaluation of risk factors for degenerative joint disease associated with hip dysplasia in German shepherd dogs, Golden retrievers, Labrador retrievers, and Rottweilers. J Am Vet Med Assoc. (2001) 219:1719–24. doi: 10.2460/javma.2001.219.1719, PMID: 11767921

[2] Smith GK Lawler DF Biery DN Powers MY Shofer F Gregor TP . Chronology of hip dysplasia development in a cohort of 48 Labrador retrievers followed for life: chronology of hip dysplasia development in Labrador retrievers. Vet Surg. (2012) 41:20–33. doi: 10.1111/j.1532-950X.2011.00935.x 23253036

[3] Riser WH Shirer JF. Correlation between canine hip dysplasia and pelvic muscle mass: a study of 95 dogs. Am J Vet Res. (1967) 28:769–77. PMID: 6068247

[4] Smith GK Popovitch CA Gregor TP Shofer FS. Evaluation of risk factors for degenerative joint disease associated with hip dysplasia in dogs. J Am Vet Med Assoc. (1995) 206:642–7. 7744684

[5] Henricson B Norberg I Olsson SE. On the etiology and pathogenesis of hip dysplasia: a comparative review. J Small Anim Pract. (1966) 7:673–88. doi: 10.1111/j.1748-5827.1966.tb04393.x, PMID: 5342030

[6] Smith GK Gregor TP Rhodes WH Biery DN. Coxofemoral joint laxity from distraction radiography and its contemporaneous and prospective correlation with laxity, subjective score, and evidence of degenerative joint disease from conventional hip-extended radiography in dogs. Am J Vet Res. (1993) 54:1021–42. doi: 10.2460/ajvr.1993.54.07.1021, PMID: 8368595

[7] Verhoeven G Fortrie R Van Ryssen B Coopman F. Worldwide screening for canine hip dysplasia: where are we now? Vet Surg. (2012) 41:10–9. doi: 10.1111/j.1532-950X.2011.00929.x, PMID: 23253035

[8] FCI online. Available online at: https://www.fci.be/en/ (accessed January 18, 2025).

[9] Smith GK Leighton EA Karbe GT McDonald-Lynch MB. Pathogenesis, diagnosis, and control of canine hip dysplasia In: Johnston SA Tobias KM, editors. Veterinary surgery small animal. 2nd ed. St. Louis, Missouri: Elsevier (2018). 964–92.

[10] Smith GK Biery DN Gregor TP. New concepts of coxofemoral joint stability and the development of a clinical stress-radiographic method for quantitating hip joint laxity in the dog. J Am Anim Hosp Assoc. (1990) 196:59–70. doi: 10.2460/javma.1990.196.01.59, PMID: 2295555

[11] Heyman SJ Smith GK Cofone MA. Biomechanical study of the effect of coxofemoral positioning on passive hip joint laxity in dogs. Am J Vet Res. (1993) 54:205–10. PMID: 8430930

[12] Farese JP Todhunter RJ Lust G Williams AJ Dykes NL. Dorsolateral subluxation of hip joints in dogs measured in a weight-bearing position with radiography and computed tomography. Vet Surg. (1998) 27:393–405. doi: 10.1111/j.1532-950x.1998.tb00146.x, PMID: 9749508

[13] Flückiger MA Friedrich GA Binder H. A radiographic stress technique for evaluation of coxofemoral joint laxity in dogs. Vet Surg. (1999) 28:1–9. doi: 10.1053/jvet.1999.0001, PMID: 10025634

[14] Smith GK LaFond E Gregor TP Lawler DF Nie RC. Within- and between-examiner repeatability of distraction indices of the hip joint in dogs. Am J Vet Res. (1997) 58:1076–7. 9328657

[15] Ginja MM Ferreira AJ Silvestre M Gonzalo-Orden JM Llorens-Pena MP. Repeatability and reproducibility of distraction indices in PennHIP examinations of the hip joint in dogs. Acta Vet Hung. (2006) 54:387–92. doi: 10.1556/AVet.54.2006.3.8, PMID: 17020141

[16] Adams WM Dueland RT Meinen J O’Brien RT Giuliano E Nordheim EV. Early detection of canine hip dysplasia: comparison of two palpation and five radiographic methods. J Am Anim Hosp Assoc. (1998) 34:339–47. doi: 10.5326/15473317-34-4-339 9657168

[17] Broeckx BJG Vezzoni A Bogaerts E Bertal M Bosmans T Stock E . Comparison of three methods to quantify laxity in the canine hip joint. Vet Comp Orthop Traumatol. (2018) 31:23–9. doi: 10.3415/VCOT17-05-0064, PMID: 29325189

[18] Bertal M De Rycke L Vezzoni A Polis I Saunders JH Broeckx BJG. Technical repeatability and reproducibility of the stress radiographs performed with the Vezzoni-modified Badertscher hip distension device. Vet Comp Orthop Traumatol. (2019) 32:67–72. doi: 10.1055/s-0038-1676306, PMID: 30646413

[19] Vezzoni A Dravelli G Corbari A De Lorenzi M Cirla A Tranquillo V. Early diagnosis of canine hip dysplasia. Eur J Comp Anim Pract. (2005) 15:173–84. 18536855

[20] Badertscher R. R.. The half-axial position: improved radiographic visualization of subluxation in canine hip dysplasia (master’s thesis). University of Georgia, Athens (GA) (1977).

[21] Vezzoni A. Early diagnosis of canine hip dysplasia (CHD). In Proceedings of 4th European FECAVA SCIVAC congress, Bologna (1998).

[22] Bertal M Vezzoni A Houdellier B Bogaerts E Stock E Polis I . Intra- and interobserver variability of measurements of the laxity index on stress radiographs performed with the Vezzoni modified Badertscher hip distension device. Vet Comp Orthop Traumatol. (2018) 31:246–51. doi: 10.1055/s-0038-1656720, PMID: 29859513

[23] Bertal M Vezzoni A Van der Vekens E Polis I Saunders JH Broeckx BJG. Analysis of a laxity index database and comparison with the Fédération Cynologique Internationale grades of this population. Vet Comp Orthop Traumatol. (2021) 34:108–14. doi: 10.1055/s-0040-1719062, PMID: 33129210

[24] Merca R Bockstahler B Vezzoni A Tichy A Boano S Vidoni B. Canine hip dysplasia screening: comparison of early evaluation to final grading in 231 dogs with Fédération Cynologique Internationale A and B. PLoS One. (2020) 15:e0233257. doi: 10.1371/journal.pone.0233257, PMID: 32421701 PMC7233575

[25] Vidoni B Aghapour M Kneissl S Vezzoni A Gumpenberger M Hechinger H . Inter-observer agreement in radiographic diagnosis of coxofemoral joint disease in a closed cohort of four-month-old rottweilers. Animals. (2022) 12:1269. doi: 10.3390/ani12101269, PMID: 35625115 PMC9137964

[26] Vezzoni A Tavola F. Diagnosi precoce della displasia di anca. Veterinaria. (2015) 29:7–39.

[27] Lust G Williams AJ Burton-Wurster N Pijanowski GJ Beck KA Rubin G . Joint laxity and its association with hip dysplasia in Labrador retrievers. Am J Vet Res. (1993) 54:1990–9. doi: 10.2460/ajvr.1993.54.12.1990, PMID: 8116927

[28] Verhoeven G Coopman F Duchateau L Saunders JH Van Rijssen B Van Bree H. Interobserver agreement in the diagnosis of canine hip dysplasia using the standard ventrodorsal hip-extended radiographic method. J Small Anim Pract. (2007) 48:387–93. doi: 10.1111/j.1748-5827.2007.00364.x, PMID: 17610468

[29] Klever J Brühschwein A Wagner S Reese S Meyer-Lindenberg A. Comparison of reliability of Norberg angle and distraction index as measurements for hip laxity in dogs. Vet Comp Orthop Traumatol. (2020) 33:274–8. doi: 10.1055/s-0040-1709460, PMID: 32349137

[30] Wilson JN Filliwuist B Garcia TC Marcellin-Little DJ. Evaluation of three acetabular measurement methods for total hip replacement in dogs. Vet Surg. (2024) 54:182–8. doi: 10.1111/vsu.14190 39503336 PMC11734875

[31] Arnaout F Dewan V Paliobeis C. The 3-dot circle: a reliable method for safe and efficient digital templating of the acetabular component. J Orthop. (2018) 15:787–91. doi: 10.1016/j.jor.2018.03.027, PMID: 30013289 PMC6043873

[32] KYON Total hip replacement online course path. Movora Education. Available online at: https://education.movora.com (accessed January 18, 2025)

[33] Dueland RT Adams WM Patricelli AJ Linn KA Crump PM. Canine hip dysplasia treated by juvenile pubic symphysiodesis: part I—two-year results. Vet Comp Orthop Traumatol. (2010) 23:306–17. doi: 10.3415/VCOT-09-04-0045 20740258

[34] Dueland RT Adams WM Patricelli AJ Linn KA Crump PM. Canine hip dysplasia treated by juvenile pubic symphysiodesis: part II—two-year results of computed tomography and distraction index. Vet Comp Orthop Traumatol. (2010) 23:318–25. doi: 10.3415/VCOT-09-04-0040 20740258

[35] Dueland RT Adams WM Fialkowski JP Patricelli AJ Mathews KG Nordheim EV. Effects of pubic symphysiodesis in dysplastic puppies. Vet Surg. (2001) 30:201–17. doi: 10.1053/jvet.2001.23350, PMID: 11340551

[36] Patricelli AJ Dueland RT Adams WM Fialkowski JP Linn KA Nordheim EV. Juvenile pubic symphysiodesis in dysplastic puppies at 15 and 20 weeks of age. Vet Surg. (2002) 31:435–44. doi: 10.1053/jvet.2002.34766, PMID: 12209414

[37] Vezzoni A Dravelli G Vezzoni L De Lorenzi M Corbari A Cirla A . Comparison of conservative management and juvenile pubic symphysiodesis in the early treatment of canine hip dysplasia. Vet Comp Orthop Traumatol. (2008) 21:267–79. doi: 10.1055/s-0037-1617372, PMID: 18536855

[38] Taroni M Genevois JP Viguier E Carozzo C Livet V Baldinger A . Evolution of radiographic parameters of canine passive hip laxity at 4, 6 and 12 months: a study of 306 dogs. Vet Comp Orthop Traumatol. (2018) 31:321–6. doi: 10.1055/s-0038-1661402, PMID: 30071569

